# Biomass, Radiation Use Efficiency, and Nitrogen Utilization of Ratoon Rice Respond to Nitrogen Management in Central China

**DOI:** 10.3389/fpls.2022.889542

**Published:** 2022-04-27

**Authors:** Chang Zheng, Yuechao Wang, Desheng Yang, Sen Xiao, Yating Sun, Jianliang Huang, Shaobing Peng, Fei Wang

**Affiliations:** National Key Laboratory of Crop Genetic Improvement, Hubei Hongshan Laboratory, MARA Key Laboratory of Crop Ecophysiology and Farming System in the Middle Reaches of the Yangtze River, College of Plant Science and Technology, Huazhong Agricultural University, Wuhan, China

**Keywords:** biomass, nitrogen management, nitrogen utilization, radiation use efficiency, ratoon rice

## Abstract

Ratoon rice is proposed as a promising way to improve rice productivity *via* increasing harvest frequency. Nitrogen (N) is the most effective in promoting the development and growth of ratoon plants. However, limited information is available on how different N management practices affect the biomass production of the ratoon crop (RC) through influencing canopy light interception, radiation use efficiency (RUE), and N utilization. Field experiments were conducted in central China in 2016 and 2017 to examine the effects of N management practices on the biomass accumulation of RC and the underlying physiological mechanisms. The N rates (100 vs. 200 kg N ha^−1^) in the main crop (MC) had a small and inconsistent effect on the biomass accumulation of RC. N application at 15 days after heading of MC for promoting bud development (N_bud_, 100 kg N ha^–1^) increased total biomass production of RC by 17.2–19.1%, due to the improvements in both pre- and post-heading biomass production during the ratoon season (BP_ratoon_). N application at 1–2 days after harvesting of MC for promoting the growth of regenerated tillers (N_tiller_, 100 kg N ha^–1^) increased total biomass production of RC by 7.8–15.9% due to the improvements in post-heading BP_ratoon_ alone or both pre- and post-heading BP_ratoon_. The differences in BP_ratoon_ caused by N_bud_ and N_tiller_ were associated with crop growth rate, leaf area index, RUE, and N uptake of RC. Total N uptake of RC was improved by N_bud_ through increasing stubble N content at harvest of MC and by N_tiller_ through increasing plant N uptake during the ratoon season. N use efficiency for BP_ratoon_ was reduced by N_tiller_ but not by N_bud_. These results suggest that both N_bud_ and N_tiller_ play important roles in improving biomass production in RC, although N_bud_ was more efficient than N_tiller_.

## Introduction

Rice (*Oryza sativa* L.) is the dominant staple food, sustaining more than 65% of the population in China (Huang and Zou, 2018). Maintaining a continuous increase in rice production is vital to meet the demand of a growing population, thus ensuring national food security (Peng et al., 2009). It has been suggested that most of this increase must come from increasing harvest frequency on the existing farmland (Ray and Foley, 2013) because it is difficult to expand farmland area and improve yield potential (Deng et al., 2019; FAOSTAT, 2020). Ratoon rice, the production of a second crop (ratoon crop, RC) developed by regenerated tillers from nodal buds on the stubble left after the first crop (main crop, MC) harvest (Harrell et al., 2009), is a promising way to boost total productivity via increasing harvest frequency (Wang et al., 2020). Without the activities of land preparation, sowing, and transplanting, the grain yield of RC can be as high as 60% of that of MC (Firouzi et al., 2018; Zheng et al., 2021). Compared with China’s other rice cropping systems, ratoon rice exhibits higher annual productivity than single-season rice and higher net economic return than double-season rice (Yuan et al., 2019). As a consequence, ratoon rice has developed rapidly in China and has been a hot research topic worldwide in recent years (Wang et al., 2021; Yu et al., 2022; Zheng et al., 2022; Zhou et al., 2022).

Many studies have focused on improving the grain yield of RC through optimizing cultural practices, especially nitrogen (N) fertilizer management (Ichii, 1988; Wang et al., 2019; Yang et al., 2021). N is the most essential nutrient element that affects the development and growth of regenerated tillers (Santos et al., 2003). It is reported that the grain yield of RC could be improved by increased N rates for both the main (N_main_) and ratoon seasons (Bahar and De Datta, 1977; Zheng et al., 2004; Xu et al., 2015). For the ratoon season, N applications for promoting bud development (N_bud_) and promoting the growth of regenerated tillers (N_tiller_) are two common N management practices, which are applied 15–20 days after the heading of MC and immediately after the harvest of MC, respectively (Xiong et al., 2000). In previous studies, the importance of N_bud_ vs. N_tiller_ for increasing the grain yield of RC has been a controversy. Some of them showed that N_bud_ had a more evident and positive influence on the grain yield of RC than N_tiller_, but the reverse was true for others (Turner and McIlrath, 1988; Yuan et al., 1996; Wang et al., 2019). Despite this discrepancy, it was proved that the yield improvement of RC by both N_bud_ and N_tiller_ was mainly attributed to aboveground total biomass production rather than the harvest index (Wang et al., 2019; Yang et al., 2021). Increasing biomass production has also been considered an important approach to further improve rice yield after the Green Revolution (Laza et al., 2003). However, limited information is available on how different N management practices affect the biomass production of RC.

Plant biomass is the product of solar radiation intercepted by the canopy and the efficiency with which the radiation is converted into biomass (radiation use efficiency, RUE) (De Costa et al., 2006). Intercepted solar radiation is determined by total incident radiation during the rice growth duration and the percentage of radiation intercepted by the canopy (Huang et al., 2016). Physiologically, plant N status influences canopy light interception through effects on canopy morphological characteristics such as leaf area, angle, and orientation (Wei et al., 2018). In addition, RUE is dependent on photosynthesis and respiration, both of which are sensitive to N uptake by rice plants (Sinclair and Horie, 1989; Reich et al., 2006). In general, high N uptake can achieve high RUE and consequently high biomass production (Li et al., 2012). In contrast, N use efficiency (NUE) has tended to decrease as the N rate has increased (Peng et al., 2006). Recent studies by Wang et al. (2019) and Yang et al. (2021) compared the effects of N_bud_ and N_tiller_ on NUE for grain production (NUE_g_) of RC and indicated that NUE_g_ of RC was reduced by N_tiller_ but not by N_bud_. However, whether the NUE for biomass production (NUE_b_) of RC could be reduced with N_main_, N_bud_, or N_tiller_ application remains unclear.

In this study, biomass accumulation, canopy light interception, RUE, N uptake, and NUE_b_ of ratoon rice were determined in a 2-year field experiment with different N treatments (N_main_, N_bud_, and N_tiller_). The objective was to examine the effects of N management on biomass accumulation of RC and its relationships with RUE and N utilization. This is the follow-up study of our previous report (Wang et al., 2019).

## Materials and Methods

### Site Description

**FIGURE 1:**
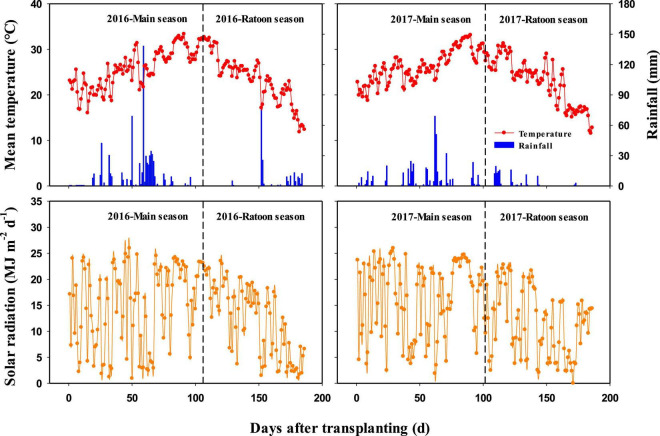
Daily mean temperature, rainfall, and solar radiation during rice growing seasons in 2016 and 2017.

Field experiments were conducted at Jiupu Village (30°23′ N, 115°43′ E), Chidong Town, Qichun County, Hubei Province, central China, during four crop growing seasons of the rice ratooning system in 2016 and 2017. Hubei Province is located in a subtropical monsoon climate zone, where ratoon rice is typically planted with MC from March to August and with RC from August to October or November. The daily mean temperature, rainfall, and solar radiation during the rice-growing seasons are shown in Figure 1. The topsoil (0–20 cm layer) of the experimental field had a clay loam texture with the following characteristics: pH 4.7, 34.5 g kg^–1^ organic matter, 2.1 g kg^–1^ total N, 11.9 mg kg^–1^ Olsen P, and 217.2 mg kg^–1^ available K.

### Experimental Design and Crop Management

The treatments were laid out in a split-split-split-plot design with four replications. The rice variety was designated as the main plot, N_main_ as the subplot, N_bud_ as the sub-subplot, and N_tiller_ as the sub-sub-subplot. Each sub-sub-subplot measured 5.0 m × 5.0 m. The varieties consisted of a hybrid Liangyou6326 (LY6326) and an inbred Huanghuazhan (HHZ), which have been widely grown for ratoon rice in the study region. For N_main_, two N levels were a moderate application rate (100 kg N ha^–1^) and a high application rate (200 kg N ha^–1^). At both N levels, N was split-applied with 40% as basal (1 day before transplanting), 30% at early tillering (7 days after transplanting), and 30% at panicle initiation in MC. For both N_bud_ and N_tiller_, two N levels were a zero-N control (0 kg N ha^–1^) and a high application rate (100 kg N ha^–1^). At a high N level, N_bud_ was applied 15 days after the heading of MC, and N_tiller_ was applied 1–2 days after the harvest of MC. All N fertilizers were applied in the form of urea. To minimize seepage between plots, all bunds were covered with plastic film inserted into the soil to form a barrier.

In both years, 38-day-old seedlings from wet bed nurseries were manually transplanted on 29 April. Transplanting was done at a hill spacing of 13.3 cm × 30.0 cm with two seedlings per hill. Before transplanting, 40 kg P ha^–1^, 60 kg K ha^–1^, and 5 kg Zn ha^–1^ were incorporated into the soil together with basal N. In addition, 60 kg K ha^–1^ was applied in all plots at the time of N_bud_ application. The sources of P, K, and Zn were calcium superphosphate, potassium chloride, and zinc sulfate heptahydrate, respectively. The MC was harvested manually on August 12, 2016 and August 09, 2017, with a stubble height of 45 cm. Details of other crop management, such as irrigation, disease, pest, and weed, were described by Wang et al. (2019). The growth durations for two varieties grown in MC and RC are provided in Supplementary Table 1.

### Sampling and Measurements

In each plot, 12 hills were sampled at the heading and maturity of both MC and RC. At the heading, the green leaf area was measured using a leaf area meter (LI-3100, LI-COR Inc., Lincoln, NE, United States) to determine the leaf area index. The dry weight of whole plants was measured after oven-drying at 80°C to constant weight to determine pre-heading biomass production. At the maturity of MC, plants were cut into lower and upper parts, which included the tissues below and above the stubble height (45 cm), respectively. For the lower part, the regenerated buds that developed during the main season were stripped out from each node of stubble. The upper part was further divided into straw and panicle. Eventually, plant samples were separated into stubble, regenerated bud, straw, and panicle. At the maturity of RC, plant samples were separated into stubble (left in MC), straw (regenerated in RC), and panicle. The dry weight of each component was measured after oven-drying at 80°C to a constant weight. Total biomass production was the total dry weight of stubble, regenerated bud (only for MC), straw, and panicle. For MC, post-heading biomass production was the difference between total biomass production and pre-heading biomass production. Pre- and post-heading crop growth rates were calculated as the ratio of biomass production to growth duration from transplanting to heading and from heading to harvest, respectively. For RC, biomass production during the ratoon season (BP_ratoon_), pre- and post-heading BP_ratoon_, and pre- and post-heading crop growth rates were calculated according to the following formulas:


(1) $${\text{BP}}_{\text{ratoon}}=\mathrm{Total}\,\mathrm{biomass}\,\mathrm{production}\,\mathrm{of}\,\mathrm{RC}-\mathrm{Dry}\,\mathrm{weights}\,\mathrm{of}\,\mathrm{stubble}\,\mathrm{and}\,\mathrm{regenerated}\,\mathrm{bud}\,\mathrm{at}\,\mathrm{harvest}\,\mathrm{of}\,\mathrm{MC}$$

(2) $$\text{Pre-heading}\,{\mathrm{BP}}_{\text{ratoon}}=\mathrm{Total}\,\mathrm{dry}\,\mathrm{weight}\,\mathrm{at}\,\mathrm{heading}\,\mathrm{of}\,\mathrm{RC}-\mathrm{Dry}\,\mathrm{weights}\,\mathrm{of}\,\mathrm{stubble}\,\mathrm{and}\,\mathrm{regenerated}\,\mathrm{bud}\,\mathrm{at}\,\mathrm{harvest}\,\mathrm{of}\,\mathrm{MC}$$

(3) $$\text{Post-heading}\,{\mathrm{BP}}_{\text{ratoon}}={\text{BP}}_{\text{ratoon}}-\text{Pre-heading}\,{\mathrm{BP}}_{\text{ratoon}}$$

(4) $$\text{Pre-heading}\mathrm{crop}\mathrm{growth}\mathrm{rate}=\text{Pre-heading}{\mathrm{BP}}_{\text{ratoon}}/\mathrm{Growth}\,\mathrm{duration}\,\mathrm{from}\,\mathrm{the}\,\mathrm{harvest}\,\mathrm{of}\,\mathrm{MC}\,\mathrm{to}\,\mathrm{the}\,\mathrm{heading}\,\mathrm{of}\,\mathrm{RC}$$

(5) $$\text{Post-heading}\mathrm{crop}\mathrm{growth}\mathrm{rate}=\text{Post-heading}{\mathrm{BP}}_{\text{ratoon}}/\mathrm{Growth}\,\mathrm{duration}\,\mathrm{from}\,\mathrm{heading}\,\mathrm{of}\,\mathrm{RC}\,\mathrm{to}\,\mathrm{harvest}\,\mathrm{of}\,\mathrm{RC}$$

The samples of each component at maturity of MC and RC were ground to measure N concentration using an elemental analyzer (Elementar Vario MAX CNS/CN, Elementar Trading Co., Ltd., Germany). The N content of each component was calculated as the product of N concentration and dry weight. Total N uptake (TNU) at maturity was the sum of the N content of each component. The N uptake during the ratoon season (NU_ratoon_) was the difference between TNU of RC and N contents of stubble and regenerated bud at harvest of MC. The NUE_b_ was calculated as the ratio of total biomass production to TNU for MC and the ratio of BP_ratoon_ to NU_ratoon_ for RC.

Canopy light interception was measured during the main (from transplanting to harvest of MC) and ratoon seasons (from harvest of MC to harvest of RC). The measurements were performed between 1,100 and 1,300 h at an interval of 7–15 days using a linear photosynthetically active radiation ceptometer (AccuPAR LP-80, Decagon Devices Inc., Pullman, WA, United States). In each plot, light intensity inside the canopy was measured by placing the light bar in the middle of two rows, slightly above the water surface. Three readings were taken between rows and another three within rows. Light intensity above the canopy was immediately recorded after the light measurement inside the canopy. Canopy light interception was calculated as the percentage of light intercepted by the canopy [100 × (light intensity above the canopy − light intensity below the canopy)/light intensity above the canopy]. Intercepted radiation during a growth period was calculated using the average canopy light interception and accumulated incident solar radiation during this growth period [1/2 × (canopy light interception at the beginning of the growth period + canopy light interception at the end of the growth period) × accumulated incident solar radiation during the growth period]. The intercepted radiation during the entire growing season was the summation of IR during each growth period. The RUE during the entire growing season was calculated as the ratio of total biomass production to intercepted radiation for MC and the ratio of BP_ratoon_ to intercepted radiation for RC.

### Data Analysis

Statistical data analysis was performed using Statistix 9.0 (Analytical Software, Tallahassee, FL, United States). All data were subjected to ANOVA using a linear model (general AOV/AOCV). ANOVA was conducted separately for each year, and individual effects of variety and N_main_ and their interactions were detected for MC, and individual effects of variety, N_main_, N_bud_, and N_tiller_ and all possible interactions were detected for RC. Mean separation was run with Tukey’s honestly significant difference (HSD) test at the 0.05 probability level. Regression analysis was conducted in a linear regression procedure to determine the coefficient of determination (*R*^2^). All graphical representations of data were performed using SigmaPlot 12.5 (Systat Software Inc., Point Richmond, CA, United States).

## Results

### Biomass Accumulation and Crop Growth Rate of Main Crop

Total biomass production of MC was significantly affected by N_main_ in both years (Table 1 and Supplementary Table 2). Compared with a moderate N rate, a high N rate increased total biomass production by 8.7% on average across varieties and years. This increase was attributed to both higher pre- and post-heading biomass production, although the differences in these two traits between N rates were insignificant in most cases. Similarly, a high N rate increased pre- and post-heading crop growth rates, thus increasing the total crop growth rate over the entire growing season.

**TABLE 1:** Biomass production and crop growth rate for two varieties grown in the main crop of 2016 and 2017.

Year	Variety	N_main_	Biomass production (g m^–2^)			Crop growth rate (g m^–2^ d^–1^)		
				
		(kg ha^–1^)	Pre-heading	Post-heading	Total	Pre-heading	Post-heading	Total
2016	LY6326	100	702.3 c	705.3 a	1407.6 c	10.5 b	18.6 ab	13.4 c
		200	757.9 b	757.1 a	1515.0 ab	11.3 a	19.9 ab	14.4 ab
		**Mean**	**730.1 B**	**731.2 A**	**1461.3 A**	**10.9 B**	**19.2 A**	**13.9 A**
	HHZ	100	859.2 a	579.0 b	1438.2 bc	11.8 a	18.1 b	13.7 bc
		200	897.8 a	655.9 ab	1553.7 a	12.3 a	20.5 a	14.8 a
		**Mean**	**878.5 A**	**617.5 B**	**1496.0 A**	**12.0 A**	**19.3 A**	**14.2 A**
2017	LY6326	100	1004.0 ab	617.0 a	1621.0 b	14.8 ab	18.1 b	15.9 b
		200	1090.7 a	693.1 a	1783.8 a	16.0 a	20.4 a	17.5 a
		**Mean**	**1047.4 A**	**655.0 A**	**1702.4 A**	**15.4 A**	**19.3 A**	**16.7 A**
	HHZ	100	961.8 b	631.8 a	1593.6 b	14.2 b	18.6 ab	15.6 b
		200	1038.9 ab	698.0 a	1736.9 a	15.3 ab	20.5 a	17.0 a
		**Mean**	**1000.3 A**	**664.9 A**	**1665.2 B**	**14.7 A**	**19.6 A**	**16.3 B**

*Within a column for each year, means followed by the same letters are not significantly different according to Tukey’s HSD test (P < 0.05). Lowercase and uppercase letters indicate comparisons among four varieties and N_main_ combinations and between two varieties, respectively.*

*N_main_, N rates for the main crop; LY6326, Liangyou6326; HHZ, Huanghuazhan.*

### Leaf Area Index, Intercepted Radiation, and Radiation Use Efficiency of Main Crop

The leaf area index at the heading was significantly increased by a high N rate of N_main_ except for HHZ in 2016 (Table 2). The high N rate also increased total intercepted radiation, and the difference between N rates was significant in 2016 but not in 2017. This increase was attributed to both higher pre- and post-heading intercepted radiation. No significant differences between N rates were observed in RUE in each growth period.

**TABLE 2:** Leaf area index at heading, intercepted radiation, and radiation use efficiency (RUE) for two varieties grown in the main crop of 2016 and 2017.

Year	Variety	N_main_	Leaf area	Intercepted radiation (MJ m^–2^)			RUE (g MJ^–1^)		
					
		(kg ha^–1^)	index	Pre-heading	Post-heading	Total	Pre-heading	Post-heading	Total
2016	LY6326	100	3.97 b	463 bc	642 b	1105 b	1.52 a	1.10 a	1.27 a
		200	4.56 a	487 b	673 a	1160 a	1.56 a	1.12 a	1.31 a
		**Mean**	**4.27 B**	**475 B**	**657 A**	**1132 A**	**1.54 A**	**1.11 A**	**1.29 A**
	HHZ	100	4.53 ab	545 ab	514 d	1059 c	1.58 a	1.13 a	1.36 a
		200	5.00 a	566 a	556 c	1122 ab	1.59 a	1.18 a	1.38 a
		**Mean**	**4.77 A**	**556 A**	**535 B**	**1091 A**	**1.58 A**	**1.16 A**	**1.37 A**
2017	LY6326	100	5.25 bc	584 b	601 b	1185 ab	1.72 a	1.03 a	1.37 a
		200	7.11 a	611 a	629 a	1240 a	1.79 a	1.10 a	1.44 a
		**Mean**	**6.18 A**	**598 A**	**615 A**	**1213 A**	**1.76 A**	**1.07 A**	**1.40 A**
	HHZ	100	4.71 c	560 c	610 b	1170 b	1.72 a	1.04 a	1.36 a
		200	5.94 b	573 bc	642 a	1215 ab	1.81 a	1.09 a	1.43 a
		**Mean**	**5.32 B**	**567 B**	**626 A**	**1193 A**	**1.77 A**	**1.07 A**	**1.40 A**

*Within a column for each year, means followed by the same letters are not significantly different according to Tukey’s HSD test (P < 0.05). Lowercase and uppercase letters indicate comparisons among four varieties and N_main_ combinations and between two varieties, respectively.*

*N_main_, N rates for the main crop; LY6326, Liangyou6326; HHZ, Huanghuazhan.*

### N Uptake and N Use Efficiency of Main Crop

Total N uptake was significantly increased by the high N rate of N_main_, and the increase was 12.8% on average across varieties and years (Table 3). In contrast, NUE_b_ was consistently decreased by a high N rate, although the difference between N rates was significant only for LY6326 in 2016.

**TABLE 3:** Total N uptake (TNU) and N use efficiency for biomass production (NUE_b_) for two varieties grown in the main crop of 2016 and 2017.

Year	Variety	N_main_	TNU	NUE_b_
				
		(kg ha^–1^)	(kg kg^–1^)	(kg kg^–1^)
2016	LY6326	100	170.0 b	82.8 a
		200	197.5 a	76.7 b
		**Mean**	**183.8 A**	**79.8 A**
	HHZ	100	172.3 b	83.5 a
		200	193.6 a	80.3 ab
		**Mean**	**183.0 A**	**81.9 A**
2017	LY6326	100	191.7 b	84.6 a
		200	215.5 a	82.8 a
		**Mean**	**203.6 A**	**83.7 A**
	HHZ	100	192.3 b	82.9 a
		200	211.6 a	82.1 a
		**Mean**	**202.0 A**	**82.5 A**

*Within a column for each year, means followed by the same letters are not significantly different according to Tukey’s HSD test (P < 0.05). Lowercase and uppercase letters indicate comparisons among four varieties and N_main_ combinations and between two varieties, respectively.*

*N_main_, N rates for the main crop; LY6326, Liangyou6326; HHZ, Huanghuazhan.*

### Related Traits of Left Stubble and Regenerated Bud at Harvest of Main Crop

**FIGURE 2:**
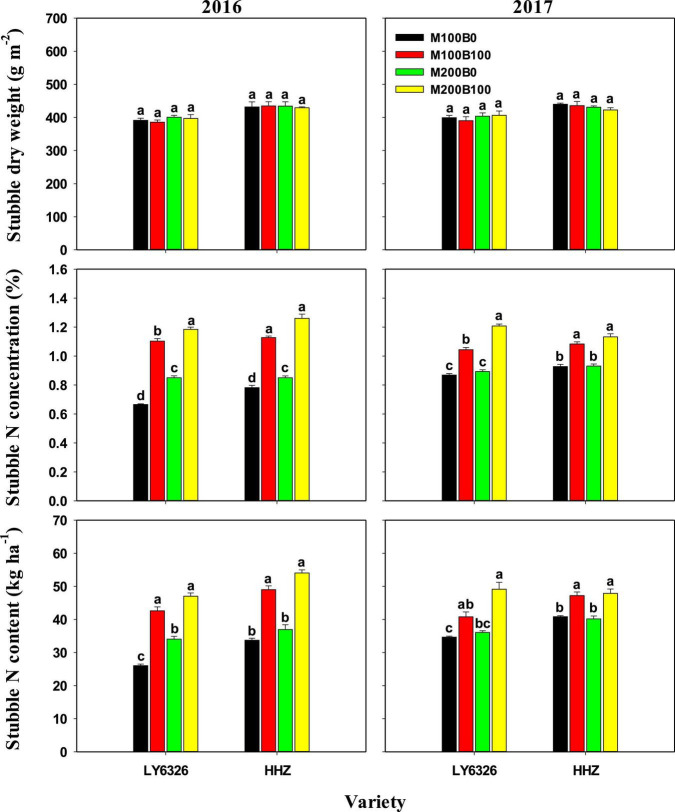
Stubble dry weight and N concentration and content at harvest of the main crop in 2016 and 2017. For each variety, means (±SE) followed by the same letters are not significantly different according to Tukey’s HSD test (*P* < 0.05). M100 and M200, N_main_ of 100 and 200 kg N ha^–1^, respectively; B0 and B100, N_bud_ of 0 and 100 kg N ha^–1^, respectively. LY6326, Liangyou6326; HHZ, Huanghuazhan.

N_main_ had small and inconsistent effects on dry weight, N concentration, and N content of left stubble at harvest of MC (Figure 2). Significant differences between N_bud_ treatments were observed in stubble N concentration and content but not in stubble dry weight. With N_bud_ application, stubble N concentration and content were increased by 49.0 and 47.9%, respectively, in 2016, and by 23.4 and 21.9%, respectively, in 2017.

**FIGURE 3:**
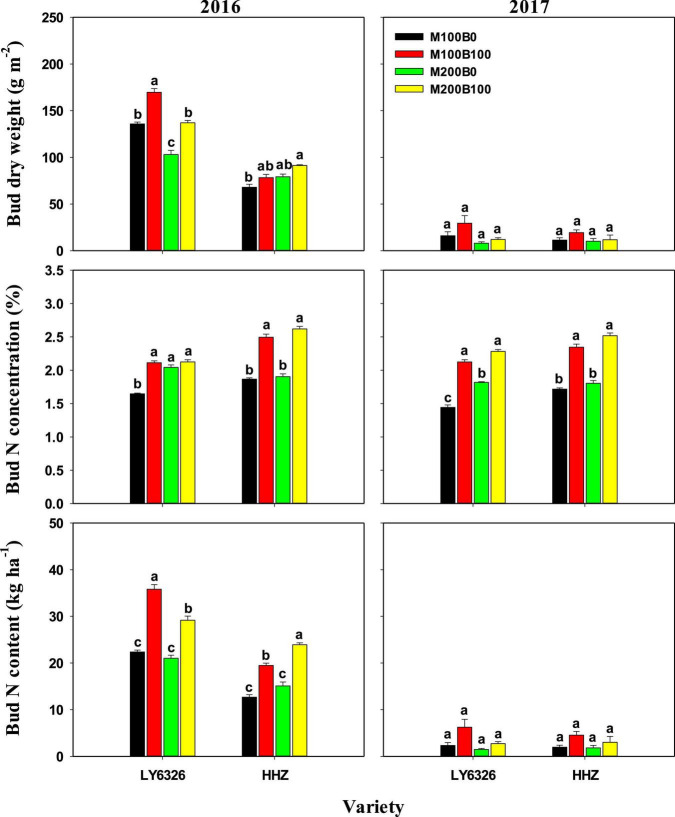
Bud dry weight and N concentration and content at harvest of the main crop in 2016 and 2017. For each variety, means (±SE) followed by the same letters are not significantly different according to Tukey’s HSD test (*P* < 0.05). M100 and M200, N_main_ of 100 and 200 kg N ha^–1^, respectively; B0 and B100, N_bud_ of 0 and 100 kg N ha^–1^, respectively. LY6326, Liangyou6326; HHZ, Huanghuazhan.

N_main_ also had small and inconsistent effects on dry weight, N concentration, and N content of regenerated bud at harvest of MC (Figure 3). Significant differences between N_bud_ treatments were observed in all three traits in 2016. In 2017, a consistent difference between N_bud_ treatments was observed only for bud N concentration. With N_bud_ application, bud dry weight, N concentration, and N content were increased by 23.5, 25.6, and 52.3%, respectively, in 2016. In 2017, N_bud_ application increased bud N concentration by 37.0%. Due to the decreased bud dry weight, bud N content was much lower in 2017 compared with 2016, whereas there was no difference in bud N concentration between 2 years.

### Biomass Accumulation and Crop Growth Rate of Ratoon Crop

Total biomass production of RC was significantly affected by all experimental factors (i.e., N_main_, N_bud_, N_tiller_, and variety) in both years (Supplementary Tables 3, 4). The high N rate of N_main_ increased total biomass production by 13.5% and 8.7% for LY6326 in 2016 and 2017, respectively, but N_main_ had a small effect on total biomass production for HHZ in both years (Tables 4, 5). Both N_bud_ and N_tiller_ applications increased total biomass production, although the effect of N_bud_ on total biomass production was more consistent than that of N_tiller_ across years. On average, N_bud_ increased total biomass production by 17.2 and 19.1% in 2016 and 2017, respectively, and N_tiller_ increased total biomass production by 7.8 and 15.9% in 2016 and 2017, respectively. Overall, N_bud_ had the largest effect on total biomass production of RC, followed by N_tiller_ and N_main_.

**TABLE 4:** Total biomass production (TBP), biomass production during ratoon season (BP_ratoon_), and crop growth rate for two varieties grown in ratoon crop of 2016.

Variety	N treatment (kg ha^–1^)			TBP	BP_ratoon_ (g m^–2^)			Crop growth rate (g m^–2^ d^–1^)		
				
	N_main_	N_bud_	N_tiller_	(g m^–2^)	Pre-heading	Post-heading	Total	Pre-heading	Post-heading	Total
LY6326	100	0	0	888.7 b	202.6 a	155.3 a	357.9 b	7.5 a	2.9 a	4.4 c
			100	999.7 a	267.9 a	208.3 a	476.2 a	9.9 a	3.9 a	5.9 b
			**Mean**	**944.2 B**	**235.3 B**	**181.8 A**	**417.1 B**	**8.7 B**	**3.4 A**	**5.2 B**
		100	0	1061.2 a	292.4 a	225.5 a	517.9 a	10.8 a	4.2 a	6.4 ab
			100	1129.2 a	301.8 a	259.1 a	560.9 a	11.2 a	4.8 a	6.9 a
			**Mean**	**1095.2 A**	**297.1 A**	**242.3 A**	**539.4 A**	**11.0 A**	**4.5 A**	**6.7 A**
	200	0	0	1021.7 c	327.5 b	198.7 a	526.2 c	12.2 b	3.7 a	6.5 c
			100	1074.7 c	320.1 b	243.2 a	563.3 c	11.9 b	4.5 a	7.0 c
			**Mean**	**1048.2 B**	**323.8 B**	**220.9 B**	**544.7 B**	**12.0 B**	**4.1 B**	**6.7 B**
		100	0	1193.2 b	371.5 ab	288.3 a	659.4 b	13.8 ab	5.3 a	8.2 b
			100	1341.7 a	493.7 a	313.6 a	807.3 a	18.3 a	5.8 a	10.0 a
			**Mean**	**1267.4 A**	**432.4 A**	**300.9 A**	**733.3 A**	**16.0 A**	**5.6 A**	**9.1 A**
HHZ	100	0	0	782.7 b	151.0 a	124.7 a	275.7 b	7.2 a	2.8 a	4.2 b
			100	877.0 ab	178.4 a	205.8 a	384.2 ab	8.5 a	4.6 a	5.8 ab
			**Mean**	**829.9 B**	**164.7 B**	**165.3 A**	**330.0 B**	**7.9 B**	**3.7 A**	**5.0 B**
		100	0	952.0 a	247.9 a	194.7 a	442.6 ab	11.8 a	4.4 a	6.7 ab
			100	976.9 a	243.8 a	216.0 a	459.8 a	11.6 a	4.8 a	7.0 a
			**Mean**	**964.5 A**	**245.9 A**	**205.3 A**	**451.2 A**	**11.7 A**	**4.6 A**	**6.8 A**
	200	0	0	845.2 b	182.2 b	156.7 a	338.9 a	8.7 b	3.5 a	5.2 b
			100	907.1 ab	173.8 b	212.8 a	386.6 a	8.3 b	4.7 a	5.9 b
			**Mean**	**876.2 B**	**178.0 B**	**184.8 A**	**362.8 A**	**8.5 B**	**4.1 A**	**5.5 B**
		100	0	988.1 ab	308.2 a	179.1 a	487.3 a	14.7 a	4.0 a	7.4 a
			100	1028.0 a	271.2 a	216.8 a	488.0 a	12.9 a	4.8 a	7.4 a
			**Mean**	**1008.1 A**	**289.7 A**	**197.9 A**	**487.6 A**	**13.8 A**	**4.4 A**	**7.4 A**

*Within a column for each N_main_ treatment in each variety, means followed by the same letters are not significantly different according to Tukey’s HSD test (P < 0.05). Lowercase and uppercase letters indicate comparisons among four N_bud_ and N_tiller_ combinations and between two N_bud_ treatments, respectively.*

*N_main_, N rates for the main crop; N_bud_, N rates for promoting bud development; N_tiller_, N rates for promoting the growth of regenerated tillers; LY6326, Liangyou6326; HHZ, Huanghuazhan.*

**TABLE 5:** Total biomass production (TBP), biomass production during ratoon season (BP_ratoon_), and crop growth rate for two varieties grown in ratoon crop of 2017.

Variety	N treatment (kg ha^–1^)			TBP	BP_ratoon_ (g m^–2^)			Crop growth rate (g m^–2^ d^–1^)		
				
	N_main_	N_bud_	N_tiller_	(g m^–2^)	Pre-heading	Post-heading	Total	Pre-heading	Post-heading	Total
LY6326	100	0	0	943.7 c	355.4 c	175.3 a	530.7 c	8.9 c	4.0 a	6.3 c
			100	1169.4 b	491.2 b	260.4 a	751.6 b	12.3 b	5.9 a	9.0 b
			**Mean**	**1056.5 B**	**423.3 B**	**217.8 A**	**641.1 B**	**10.6 B**	**5.0 A**	**7.7 B**
		100	0	1156.9 b	487.6 bc	235.8 a	723.4 b	12.2 bc	5.4 a	8.6 b
			100	1351.5 a	667.0 a	278.2 a	945.2 a	16.7 a	6.3 a	11.3 a
			**Mean**	**1254.2 A**	**577.3 A**	**257.0 A**	**834.3 A**	**14.4 A**	**5.8 A**	**9.9 A**
	200	0	0	994.4 c	386.9 c	198.9 a	585.8 c	9.7 c	4.5 a	7.0 c
			100	1277.9 b	590.9 b	271.0 a	861.9 b	14.8 b	6.2 a	10.2 b
			**Mean**	**1136.2 B**	**488.9 B**	**235.0 A**	**723.9 B**	**12.2 B**	**5.3 A**	**8.6 B**
		100	0	1299.4 b	593.3 b	291.4 a	884.7 b	14.8 b	6.6 a	10.5 b
			100	1452.9 a	709.5 a	321.2 a	1030.7 a	17.8 a	7.3 a	12.3 a
			**Mean**	**1376.2 A**	**651.4 A**	**306.3 A**	**957.7 A**	**16.3 A**	**7.0 A**	**11.4 A**
HHZ	100	0	0	828.6 d	225.3 b	142.4 b	367.7 c	7.5 b	4.6 b	6.0 c
			100	960.4 c	299.5 ab	219.2 a	518.7 b	10.0 ab	7.1 a	8.5 b
			**Mean**	**894.5 B**	**262.4 B**	**180.8 B**	**443.2 B**	**8.8 B**	**5.8 B**	**7.3 B**
		100	0	1020.8 b	334.2 a	233.9 a	568.1 ab	11.1 a	7.6 a	9.3 ab
			100	1117.0 a	385.0 a	274.2 a	659.2 a	12.8 a	8.9 a	10.8 a
			**Mean**	**1068.9 A**	**359.6 A**	**254.0 A**	**613.6 A**	**12.0 A**	**8.2 A**	**10.1 A**
	200	0	0	846.3 d	239.3 c	165.7 b	405.0 c	8.0 c	5.4 b	6.7 c
			100	972.6 c	297.9 b	233.5 ab	531.4 b	10.0 b	7.5 ab	8.7 b
			**Mean**	**909.5 B**	**268.6 B**	**199.6 B**	**468.2 B**	**9.0 B**	**6.4 B**	**7.7 B**
		100	0	1020.9 b	340.8 b	233.8 a	574.6 b	11.4 b	7.6 a	9.4 b
			100	1098.1 a	397.3 a	277.8 a	675.1 a	13.2 a	9.0 a	11.1 a
			**Mean**	**1059.5 A**	**369.0 A**	**255.8 A**	**624.8 A**	**12.3 A**	**8.3 A**	**10.2 A**

*Within a column for each N_main_ treatment in each variety, means followed by the same letters are not significantly different according to Tukey’s HSD test (P < 0.05). Lowercase and uppercase letters indicate comparisons among four N_bud_ and N_tiller_ combinations and between two N_bud_ treatments, respectively.*

*N_main_, N rates for the main crop; N_bud_, N rates for promoting bud development; N_tiller_, N rates for promoting the growth of regenerated tillers; LY6326, Liangyou6326; HHZ, Huanghuazhan.*

The effect of N_main_ on total BP_ratoon_ was small and inconsistent, and total BP_ratoon_ was mainly affected by N_bud_ and N_tiller_ (Tables 4, 5). With N_bud_ application, total BP_ratoon_ was increased by 33.6 and 33.1% in 2016 and 2017, respectively. The application of N_tiller_ also increased total BP_ratoon_, but the improvement in 2016 (14.4%) was smaller than that in 2017 (28.7%). Both pre- and post-heading BP_ratoon_ contributed to the increase in total BP_ratoon_ by N_bud_, although the contribution from pre-heading BP_ratoon_ was greater than that from post-heading BP_ratoon_. The increase in total BP_ratoon_ by N_tiller_ was explained by post-heading BP_ratoon_ alone in 2016 and by both pre- and post-heading BP_ratoon_ in 2017. The effects of N_bud_ and N_tiller_ on crop growth rate were similar to those on BP_ratoon_.

### Leaf Area Index, Intercepted Radiation, and Radiation Use Efficiency of Ratoon Crop

N_main_ had small and inconsistent effects on leaf area index at heading, intercepted radiation, and RUE. The application of N_bud_ significantly and consistently increased leaf area index across the years, whereas the effect of N_tiller_ on leaf area index was significant in 2017 but not in 2016 (Tables 6, 7). Even though both N_bud_ and N_tiller_ applications increased intercepted radiation in each growth period, all the improvements were relatively small. Compared with N_tiller_, N_bud_ had a more consistent effect on total RUE across years. With N_bud_ application, total RUE was increased by 28.1 and 29.5% in 2016 and 2017, respectively, resulting from the increases in both pre- and post-heading RUE. With N_tiller_ application, total RUE was increased by 7.9 and 23.3% in 2016 and 2017, resulting from the increases in post-heading RUE alone and both pre- and post-heading RUE, respectively.

**TABLE 6:** Leaf area index at heading, intercepted radiation, and radiation use efficiency (RUE) for two varieties grown in ratoon crop of 2016.

Variety	N treatment (kg ha^–1^)			Leaf area	Intercepted radiation (MJ m^–2^)			RUE (g MJ^–1^)		
				
	N_main_	N_bud_	N_tiller_	index	Pre-heading	Post-heading	Total	Pre-heading	Post-heading	Total
LY6326	100	0	0	1.10 c	287 a	439 a	726 b	0.71 b	0.35 a	0.50 a
			100	1.27 b	309 a	470 a	779 ab	0.87 ab	0.44 a	0.61 a
			**Mean**	**1.18 B**	**298 A**	**454 A**	**752 B**	**0.79 B**	**0.40 A**	**0.55 A**
		100	0	2.22 a	305 a	462 a	767 ab	0.96 a	0.49 a	0.68 a
			100	2.23 a	325 a	474 a	799 a	0.93 a	0.55 a	0.71 a
			**Mean**	**2.22 A**	**315 A**	**468 A**	**783 A**	**0.95 A**	**0.52 A**	**0.69 A**
	200	0	0	1.38 c	296 a	461 a	757 a	1.11 b	0.43 a	0.70 c
			100	1.89 b	316 a	477 a	793 a	1.01 b	0.51 a	0.71 c
			**Mean**	**1.63 B**	**306 A**	**469 A**	**775 A**	**1.06 B**	**0.47 B**	**0.70 B**
		100	0	2.13 ab	316 a	470 a	786 a	1.18 b	0.61 a	0.84 b
			100	2.41 a	329 a	485 a	814 a	1.50 a	0.65 a	0.99 a
			**Mean**	**2.27 A**	**323 A**	**477 A**	**800 A**	**1.34 A**	**0.63 A**	**0.92 A**
HHZ	100	0	0	0.90 b	214 a	423 b	637 b	0.71 b	0.29 a	0.43 a
			100	0.89 b	229 a	479 a	708 a	0.78 b	0.43 a	0.55 a
			**Mean**	**0.90 B**	**221 A**	**451 A**	**672 A**	**0.73 B**	**0.36 A**	**0.49 B**
		100	0	1.23 a	237 a	458 ab	695 a	1.05 a	0.43 a	0.64 a
			100	1.28 a	233 a	476 a	709 a	1.05 a	0.45 a	0.65 a
			**Mean**	**1.26 A**	**235 A**	**472 A**	**702 A**	**1.05 A**	**0.44 A**	**0.64 A**
	200	0	0	1.12 b	219 a	436 b	655 a	0.83 bc	0.36 a	0.52 a
			100	1.19 b	230 a	485 a	715 a	0.76 c	0.44 a	0.54 a
			**Mean**	**1.15 B**	**225 A**	**460 A**	**685 A**	**0.79 B**	**0.40 A**	**0.53 A**
		100	0	1.26 b	239 a	470 a	709 a	1.29 a	0.38 a	0.69 a
			100	1.52 a	232 a	485 a	717 a	1.17 ab	0.45 a	0.68 a
			**Mean**	**1.39 A**	**236 A**	**477 A**	**713 A**	**1.20 A**	**0.41 A**	**0.68 A**

*Within a column for each N_main_ treatment in each variety, means followed by the same letters are not significantly different according to Tukey’s HSD test (P < 0.05). Lowercase and uppercase letters indicate comparisons among four N_bud_ and N_tiller_ combinations and between two N_bud_ treatments, respectively.*

*N_main_, N rates for the main crop; N_bud_, N rates for promoting bud development; N_tiller_, N rates for promoting the growth of regenerated tillers; LY6326, Liangyou6326; HHZ, Huanghuazhan.*

**TABLE 7:** Leaf area index at heading, intercepted radiation, and radiation use efficiency (RUE) for two varieties grown in ratoon crop of 2017.

Variety	N treatment (kg ha^–1^)			Leaf area	Intercepted radiation (MJ m^–2^)			RUE (g MJ^–1^)		
				
	N_main_	N_bud_	N_tiller_	Index	Pre-heading	Post-heading	Total	Pre-heading	Post-heading	Total
LY6326	100	0	0	2.18 c	378 a	373 a	751 a	0.94 c	0.47 a	0.71 c
			100	4.13 ab	394 a	401 a	795 a	1.25 b	0.65 a	0.95 ab
			**Mean**	**3.15 B**	**386 A**	**387 A**	**773 A**	**1.09 B**	**0.56 A**	**0.83 B**
		100	0	3.69 b	389 a	396 a	785 a	1.25 b	0.60 a	0.92 bc
			100	4.85 a	404 a	412 a	816 a	1.65 a	0.68 a	1.16 a
			**Mean**	**4.27 A**	**396 A**	**404 A**	**800 A**	**1.45 A**	**0.64 A**	**1.04 A**
	200	0	0	2.75 c	334 a	395 a	729 a	1.16 b	0.50 a	0.81 b
			100	4.53 b	352 a	411 a	763 a	1.68 a	0.66 a	1.13 a
			**Mean**	**3.64 B**	**343 A**	**403 A**	**746 A**	**1.42 B**	**0.58 A**	**0.97 B**
		100	0	3.33 c	344 a	409 a	753 a	1.72 a	0.71 a	1.18 a
			100	5.34 a	356 a	408 a	764 a	1.99 a	0.79 a	1.35 a
			**Mean**	**4.34 A**	**350 A**	**408 A**	**758 A**	**1.86 A**	**0.75 A**	**1.26 A**
HHZ	100	0	0	1.12 c	229 a	267 b	496 b	0.98 b	0.53 b	0.74 c
			100	2.21 ab	252 a	301 a	553 ab	1.19 ab	0.73 ab	0.95 bc
			**Mean**	**1.67 B**	**241 A**	**283 A**	**524 B**	**1.08 B**	**0.63 B**	**0.84 B**
		100	0	2.01 b	245 a	297 a	542 ab	1.36 ab	0.79 a	1.05 ab
			100	2.31 a	254 a	303 a	557 a	1.52 a	0.90 a	1.19 a
			**Mean**	**2.16 A**	**250 A**	**299 A**	**549 A**	**1.44 A**	**0.85 A**	**1.12 A**
	200	0	0	1.35 c	239 a	288 a	527 a	1.00 c	0.58 b	0.77 c
			100	2.41 a	253 a	304 a	557 a	1.18 bc	0.77 ab	0.96 b
			**Mean**	**1.88 B**	**246 A**	**296 A**	**542 B**	**1.09 B**	**0.68 B**	**0.86 B**
		100	0	1.98 b	250 a	300 a	550 a	1.36 ab	0.78 ab	1.05 b
			100	2.44 a	259 a	302 a	561 a	1.53 a	0.92 a	1.21 a
			**Mean**	**2.21 A**	**254 A**	**289 A**	**556 A**	**1.45 A**	**0.85 A**	**1.13 A**

*Within a column for each N_main_ treatment in each variety, means followed by the same letters are not significantly different according to Tukey’s HSD test (P < 0.05). Lowercase and uppercase letters indicate comparisons among four N_bud_ and N_tiller_ combinations and between two N_bud_ treatments, respectively.*

*N_main_, N rates for the main crop; N_bud_, N rates for promoting bud development; N_tiller_, N rates for promoting the growth of regenerated tillers; LY6326, Liangyou6326; HHZ, Huanghuazhan.*

### N Uptake and N Use Efficiency of Ratoon Crop

N_main_ had small and inconsistent effects on N uptake and NUE of RC. Both N_bud_ and N_tiller_ had significant effects on TNU in both years (Supplementary Tables 5, 6). With N_bud_ and N_tiller_ applications, TNU was increased by 27.8 and 31.8%, respectively (Tables 8, 9). The applications of N_bud_ and N_tiller_ also increased UN_ratoon_, but the effect of N_tiller_ on UN_ratoon_ was larger and more consistent than that of N_bud_ across the years. With N_tiller_ application, UN_ratoon_ was increased by 101.9 and 61.1% in 2016 and 2017, respectively. The effect of N_bud_ on the UN_ratoon_ was significant in 2017 but not in 2016. As a consequence, NU_ratoon_/TNU was mainly affected by N_tiller_ rather than N_bud_. For NUE_b_, N_bud_ and N_tiller_ had opposite effects in both years. NUE_b_ was increased with the N_bud_ application, and the effect was significant in 2016 but not in 2017. In contrast, N_tiller_ application decreased NUE_b_ by 43.9 and 20.8% in 2016 and 2017, respectively.

**TABLE 8:** Total N uptake (TNU), N uptake during ratoon season (NU_ratoon_) and its ratio to total N uptake (NU_ratoon_/TNU), and nitrogen use efficiency for biomass production during ratoon season (NUE_b_) for two varieties grown in ratoon crop of 2016.

Variety	N treatment (kg ha^–1^)			TNU	NU_ratoon_	NU_ratoon_/TNU	NUE_b_
					
	N_main_	N_bud_	N_tiller_	(kg ha^–1^)	(kg ha^–1^)	(%)	(kg kg^–1^)
LY6326	100	0	0	71.2 c	22.8 b	31.9 c	158.4 a
			100	105.0 b	56.6 a	53.9 a	84.1 b
			**Mean**	**88.1 B**	**39.7 A**	**42.9 A**	**121.3 B**
		100	0	104.0 b	25.6 b	24.6 d	204.9 a
			100	131.1 a	52.7 a	40.1 b	106.8 b
			**Mean**	**117.5 A**	**39.1 A**	**32.3 B**	**155.8 A**
	200	0	0	88.9 c	33.8 c	37.8 b	158.8 a
			100	115.6 b	60.5 b	52.3 a	93.1 b
			**Mean**	**102.2 B**	**47.1 B**	**45.1 A**	**125.9 A**
		100	0	114.4 b	38.2 c	33.4 b	172.7 a
			100	155.2 a	79.0 a	50.8 a	103.1 b
			**Mean**	**134.8 A**	**58.6 A**	**42.1 A**	**137.9 A**
HHZ	100	0	0	68.0 c	21.6 b	31.5 bc	126.7 ab
			100	95.5 b	49.1 a	51.4 a	78.7 c
			**Mean**	**81.8 B**	**35.4 A**	**41.4 A**	**102.7 B**
		100	0	94.5 b	26.0 b	27.2 c	179.3 a
			100	116.2 a	47.7 a	40.8 ab	97.2 bc
			**Mean**	**105.3 A**	**36.8 A**	**34.0 A**	**138.3 A**
	200	0	0	75.8 c	23.8 b	31.2 bc	143.3 b
			100	101.6 b	49.6 a	48.7 a	77.5 c
			**Mean**	**88.7 B**	**36.7 A**	**39.9 A**	**110.4 B**
		100	0	102.1 b	24.1 b	23.3 c	211.4 a
			100	118.8 a	40.8 a	34.2 b	120.4 bc
			**Mean**	**110.4 A**	**32.4 A**	**28.8 B**	**165.9 A**

*Within a column for each N_main_ treatment in each variety, means followed by the same letters are not significantly different according to Tukey’s HSD test (P < 0.05). Lowercase and uppercase letters indicate comparisons among four N_bud_ and N_tiller_ combinations and between two N_bud_ treatments, respectively.*

*N_main_, N rates for the main crop; N_bud_, N rates for promoting bud development; N_tiller_, N rates for promoting the growth of regenerated tillers; LY6326, Liangyou6326; HHZ, Huanghuazhan.*

**TABLE 9:** Total N uptake (TNU), N uptake during ratoon season (NU_ratoon_) and its ratio to total N uptake (NU_ratoon_/TNU), and nitrogen use efficiency for biomass production during ratoon season (NUE_b_) for two varieties grown in ratoon crop of 2017.

Variety	N treatment (kg ha^–1^)			TNU	NU_ratoon_	NU_ratoon_/TNU	NUE_b_
					
	N_main_	N_bud_	N_tiller_	(kg ha^–1^)	(kg ha^–1^)	(%)	(kg kg^–1^)
LY6326	100	0	0	83.0 c	46.0 d	55.3 b	116.5 ab
			100	118.3 b	81.3 b	68.7 a	92.5 b
			**Mean**	**100.7 B**	**63.7 B**	**62.0 A**	**104.5 B**
		100	0	109.0 b	62.0 c	56.8 b	117.5 a
			100	138.7 a	91.7 a	66.1 a	103.2 ab
			**Mean**	**123.9 A**	**76.9 A**	**61.4 A**	**110.3 A**
	200	0	0	94.4 c	56.9 d	60.2 c	103.4 b
			100	135.2 b	97.7 b	72.2 a	88.4 c
			**Mean**	**114.8 B**	**77.3 B**	**66.2 A**	**95.9 B**
		100	0	125.0 b	73.1 c	58.4 c	121.4 a
			100	162.2 a	110.3 a	67.9 b	93.8 bc
			**Mean**	**143.6 A**	**91.7 A**	**63.1 B**	**107.6 A**
HHZ	100	0	0	74.0 c	31.2 c	42.1 c	118.4 a
			100	104.7 b	61.9 b	59.0 a	83.8 bc
			**Mean**	**89.4 B**	**46.6 B**	**50.6 B**	**101.1 A**
		100	0	104.5 b	52.7 b	50.4 b	108.6 ab
			100	132.8 a	81.0 a	61.0 a	81.4 c
			**Mean**	**118.6 A**	**66.8 A**	**55.7 A**	**95.0 A**
	200	0	0	84.3 c	42.3 d	50.1 b	96.0 a
			100	113.2 b	71.2 b	62.9 a	74.6 b
			**Mean**	**98.8 B**	**56.8 B**	**56.5 B**	**85.3 A**
		100	0	108.7 b	57.8 c	53.1 b	99.6 a
			100	134.9 a	84.0 a	62.2 a	80.6 b
			**Mean**	**121.8 A**	**70.9 A**	**57.7 A**	**90.1 A**

*Within a column for each N_main_ treatment in each variety, means followed by the same letters are not significantly different according to Tukey’s HSD test (P < 0.05). Lowercase and uppercase letters indicate comparisons among four N_bud_ and N_tiller_ combinations and between two N_bud_ treatments, respectively.*

*N_main_, N rates for the main crop; N_bud_, N rates for promoting bud development; N_tiller_, N rates for promoting the growth of regenerated tillers; LY6326, Liangyou6326; HHZ, Huanghuazhan.*

### Regression Analysis

**FIGURE 4:**
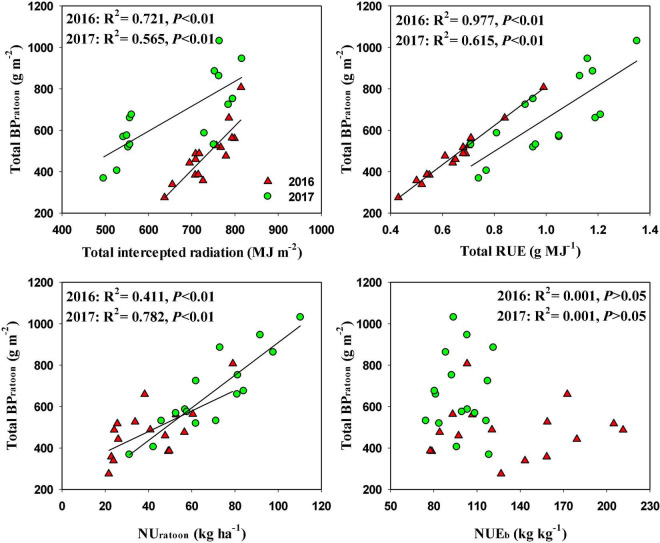
Relationships total biomass production during ratoon season (BP_*ratoon*_) and total intercepted radiation, total radiation use efficiency (RUE), N uptake during ratoon season (UN_*ratoon*_), and N use efficiency for biomass production (NUE_*b*_) in ratoon crop of 2016 and 2017.

In RC, total BP_ratoon_ was significantly and positively related to total intercepted radiation and total RUE in both years, and the coefficients of determination were higher for total RUE (*R*^2^ = 0.977 in 2016 and *R*^2^ = 0.615 in 2017) than for total intercepted radiation (*R*^2^ = 0.721 in 2016 and *R*^2^ = 0.565 in 2017) (Figure 4). Total BP_ratoon_ was also significantly and positively related to NU_ratoon_, and the coefficients of determination were 0.411 and 0.782 in 2016 and 2017, respectively. However, there was an insignificant relationship between total BP_ratoon_ and NUE_b_ in both years.

## Discussion

Rice yield is determined by biomass production and harvest index (Yoshida, 1981). However, it is widely accepted that there is less scope to further increase the harvest index after the Green Revolution (Laza et al., 2003), and achieving greater rice yield primarily depends on increasing biomass production (Peng et al., 1999). Consistently, our previous study showed that total biomass production instead of harvest index explained the differences in grain yield of RC caused by N_bud_ and N_tiller_ (Wang et al., 2019). In this study, we further proved that the positive effects of N_bud_ and N_tiller_ on total biomass production were mainly due to increased BP_ratoon_, because there were small differences in dry weights of stubble and regenerated bud at harvest of MC under different N treatments. Both pre- and post-heading BP_ratoon_ contributed to the increase in total BP_ratoon_ by N_bud_, although the contribution from pre-heading BP_ratoon_ was greater than that from post-heading BP_ratoon_. However, the increase in total BP_ratoon_ by N_tiller_ was attributed to increased post-heading BP_ratoon_ alone or both increased pre- and post-heading BP_ratoon_. Overall, the effect of N_bud_ on BP_ratoon_ was more evident and consistent than that of N_tiller_. In addition, N_main_ had an even smaller effect on BP_ratoon_ than N_tiller_.

Biomass production is a function of growth duration and crop growth rate (Yoshida, 1981). In our study, there was a similar growth duration under different N treatments for both varieties (Supplementary Table 1). The differences in BP_ratoon_ caused by N_bud_ and N_tiller_ were solely attributed to the crop growth rate. The application of N_bud_ and N_tiller_ could facilitate the development and growth of regenerated buds, thus increasing crop growth rate in the ratoon season. Besides, the high crop growth rate is largely driven by an increased leaf area index (Loomis and Connor, 1992; Ying et al., 1998). Our result showed that the leaf area index at the heading stage of RC was improved with N_bud_ and N_tiller_ applications, although the improvement by N_tiller_ was not as great as that by N_bud_.

In another approach, biomass production can be improved through increasing intercepted radiation or RUE or both (Huang et al., 2016). It is also reported that optimal N application is important to intercept more photosynthetically active radiation and obtain high RUE (Li et al., 2012). In our study, N_bud_ and N_tiller_ significantly increased RUE in the ratoon season, whereas the effects of these two N treatments on intercepted radiation were relatively small. With N_bud_ and N_tiller_ applications, total RUE was increased by 28.8 and 15.6%, respectively, and total intercepted radiation was increased by only 3.5 and 5.0%, respectively. The increase in RUE could be a result of improved canopy photosynthesis in the ratoon season with N_bud_ and N_tiller_ applications. Moreover, the regression analysis indicated that BP_ratoon_ was highly related to RUE. These results demonstrated that RUE was the key factor that determines the variation in BP_ratoon_ under different N management practices. This finding was consistent with a previous study, which stated that further improvement in biomass production in rice depended on the increased RUE rather than intercepted radiation (Mitchell and Sheehy, 2006).

N_bud_ and N_tiller_ increased TNU at the maturity of RC in different ways. For RC, plant N accumulation consisted of the N contents in the stubble and regenerated bud at harvest of MC and the N uptake by the plant during the ratoon season (NU_ratoon_). N_bud_ increased TNU mainly by increasing the N contents in the stubble and regenerated bud at harvest of MC, and partially by increasing NU_ratoon_. In contrast, N_tiller_ increased TNU by increasing NU_ratoon_. The underpinning factor for this disparity in N uptake between these two N treatments resulted from the different times of application for N_bud_ and N_tiller_. In general, N_bud_ is applied around the middle point between heading and harvest of MC, and it is not easy to be directly absorbed by RC plants. However, applied N_bud_ could accumulate in left stubble after the harvest of MC, which provides N nutrients for the development of regenerated buds (Zhang et al., 1980). Ma et al. (1992) explored the N uptake and distribution of N_bud_ in ratoon rice using ^15^N isotope identification technology and reported that the remobilization of N_bud_ remaining in left stubble played an important role in supporting the growth of RC plants. In contrast, N_tiller_ is applied after the harvest of MC, and it can be rapidly absorbed and utilized by the regenerated tillers in RC. Despite this, NUE_b_ of RC was significantly reduced by N_tiller_, which might be due to excessive N fertilizer input (Peng et al., 2010). Under the conditions of our study, N_bud_ significantly increased plant N accumulation and biomass production of RC but not reduced NUE_b_.

There was a similar stubble dry weight at harvest of MC and total biomass production in RC between 2016 and 2017. However, the BP_ratoon_ of 2016 was lower than that of 2017, resulting in a higher dry weight of regenerated buds developed during the main season. In 2016, the field was flooded with a water depth of 25–40 cm continuously for 8 days when MC was in the late stage of panicle development (Figure 1). The partial submergence limited the grain filling of MC (Lin et al., 1997), which led to more assimilates accumulated in stem and sheath that accelerated the development and growth of regenerated buds. In this case, more N_bud_ was absorbed by the regenerated buds before MC was harvested in 2016, as compared with 2017. At harvest of MC, the N content in regenerated buds ranged from 12.7 to 35.8 kg ha^–1^ in 2016, whereas that ranged from 1.5 to 6.2 kg ha^–1^ in 2017. These results also partially explained why the effects of N_bud_ on the crop growth rate, RUE, leaf area index, and BP_ratoon_ of RC were larger than those of N_tiller_ and why the effects of N_bud_ on UN_ratoon_ and NU_ratoon_/TNU of RC were smaller than those of N_tiller_ in 2016. Therefore, the importance of N_bud_ and N_tiller_ in improving the biomass accumulation and yield formation of RC not only depends on the stubble N status of MC but also is associated with the effect of the environment on the development of regenerated buds. Our findings would provide more evidence for contradictions about the effects of N_bud_ vs. N_tiller_ on plant growth of RC in different field trials.

## Conclusion

Biomass production of RC was mainly affected by N_bud_ and N_tiller_ rather than N_main_. The improvement in biomass production during the ratoon season by N_bud_ was larger than that by N_tiller_, due to more evident and consistent effects of N_bud_ on the crop growth rate, leaf area index, radiation use efficiency, and plant N uptake. NUE for biomass production during the ratoon season was reduced by N_tiller_ but not by N_bud_.

## Data Availability Statement

The original contributions presented in the study are included in the article/Supplementary Material, further inquiries can be directed to the corresponding author.

## Author Contributions

CZ collected and analyzed the data and wrote the manuscript. YW conducted the field experiments and collected and analyzed the data. DY, SX, and YS collected the data. JH provided advice on experimental implementation. SP conceived and supervised the field experiments. FW supervised the field experiments and revised the manuscript. All authors contributed to the article and approved the submitted version.

## Conflict of Interest

The authors declare that the research was conducted in the absence of any commercial or financial relationships that could be construed as a potential conflict of interest.

## Publisher’s Note

All claims expressed in this article are solely those of the authors and do not necessarily represent those of their affiliated organizations, or those of the publisher, the editors and the reviewers. Any product that may be evaluated in this article, or claim that may be made by its manufacturer, is not guaranteed or endorsed by the publisher.
