# Supplementary material for: Biomass, Radiation Use Efficiency, and Nitrogen Utilization of Ratoon Rice Respond to Nitrogen Management in Central China
Source: Front Plant Sci. 2022 Apr 27;13:889542. doi: 10.3389/fpls.2022.889542 (PMC9112333; doi:10.3389/fpls.2022.889542)
Supplement: Supplementary file 1 [file Data_Sheet_1.docx]

**Supplementary Materials**

**Table S1** Growth durations for two varieties grown in main crop (from sowing to harvest of main crop) and ratoon crop (from harvest of main crop to harvest of ratoon crop) of 2016 and 2017.

| Year | Variety | Main crop | | | |  | Ratoon crop | | |
| --- | --- | --- | --- | --- | --- | --- | --- | --- | --- |
|  |  | SW-TP | TP-MHD | MHD-MH | Total |  | MH-RHD | RHD-RH | Total |
| 2016 | LY6326 | 38 | 67 | 38 | 143 |  | 27 | 54 | 81 |
|  | HHZ | 38 | 73 | 32 | 143 |  | 21 | 45 | 66 |
| 2017 | LY6326 | 38 | 68 | 34 | 140 |  | 40 | 44 | 84 |
|  | HHZ | 38 | 68 | 34 | 140 |  | 30 | 31 | 61 |

SW, sowing; TP, transplanting; MHD, heading of the main crop; MH, harvest of the main crop; RHD, heading of the ratoon crop; RH, harvest of the ratoon crop.

The dates of each growth stage were the same under different N treatments.

**Table S2** Analysis of variance for parameters of main crop in 2016 and 2017.

| Source | Pre-BP | Post-BP | TBP | Pre-CGR | Post-CGR | Total CGR | LAI | Pre-IR | Post-IR | Total IR | Pre-RUE | Post-RUE | Total RUE | TNU | NUE_b_ |
| --- | --- | --- | --- | --- | --- | --- | --- | --- | --- | --- | --- | --- | --- | --- | --- |
| 2016 |  |  |  |  |  |  |  |  |  |  |  |  |  |  |  |
| Variety (V) | ** | ** | ns | ** | ns | ns | ** | ** | ** | * | ns | ns | ns | ns | ns |
| N_main_ | ** | ** | ** | ** | ** | ** | ** | ** | ** | ** | ns | ns | ns | ** | * |
| V×N_main_ | ns | ns | ns | ns | ns | ns | ns | ns | ** | * | ns | ns | ns | ns | ns |
|  |  |  |  |  |  |  |  |  |  |  |  |  |  |  |  |
| 2017 |  |  |  |  |  |  |  |  |  |  |  |  |  |  |  |
| Variety (V) | ns | ns | ns | ns | ns | ns | ** | ** | ** | ns | ns | ns | ns | ns | ns |
| N_main_ | ** | ns | ** | ** | ns | ** | ** | ** | ** | ** | ns | ns | ns | ** | ns |
| V×N_main_ | ns | ns | ns | ns | ns | ns | ns | ns | ns | ns | ns | ns | ns | ns | ns |

BP, biomass production; TBP, total biomass production; CGR, crop growth rate; IR, intercepted radiation; RUE, radiation use efficiency; TNU, total N uptake; NUE_b_, N use efficiency for biomass production.

ns, not significant at the 0.05 probability level; * and **, significant at the 0.05 and 0.01 probability levels, respectively.

**Table S3** Analysis of variance for biomass production, crop growth rate, leaf area index, intercepted radiation, and radiation use efficiency of ratoon crop in 2016.

| Source | TBP | Pre-BP_ratoon_ | Post-BP_ratoon_ | Total BP_ratoon_ | Pre-CGR | Post-CGR | Total CGR | LAI | Pre-IR | Post-IR | Total IR | Pre-RUE | Post-RUE | Total RUE |
| --- | --- | --- | --- | --- | --- | --- | --- | --- | --- | --- | --- | --- | --- | --- |
| Variety (V) | ** | ** | ** | ** | ** | ns | ** | ** | ** | ns | ** | * | ** | ** |
| N_main_ | ** | ** | ns | ** | ** | ns | ** | ** | ns | ns | ns | ** | ns | * |
| N_bud_ | ** | ** | ** | ** | ** | ** | ** | ** | * | * | * | ** | ** | ** |
| N_tiller_ | ** | ns | ** | ** | ns | ** | ** | ns | ns | ** | * | ns | * | * |
| V×N_main_ | ** | ** | ns | ** | ** | ns | ** | ns | ns | ns | ns | * | ns | ** |
| V×N_bud_ | ns | ns | ns | ns | ns | ns | ns | ** | * | * | ns | ns | ns | ns |
| V×N_tiller_ | ns | * | ns | ns | * | ns | ns | ** | * | * | ns | ns | ns | ns |
| N_main_×N_bud_ | ns | ns | ns | ns | ns | ns | ns | ** | ns | ns | ns | ns | ns | ns |
| N_main_×N_tiller_ | ns | ns | ns | ns | ns | ns | ns | ** | ns | ns | ns | ns | ns | ns |
| N_bud_×N_tiller_ | ns | ns | ns | ns | ns | ns | ns | ns | ns | ** | ** | ns | ns | ns |
| V×N_main_×N_bud_ | ns | ns | ns | ns | ns | ns | ns | ns | ns | ns | ns | ns | ns | ns |
| V×N_main_×N_tiller_ | ns | ns | ns | ns | ns | ns | ns | ns | ns | ns | ns | ns | ns | ns |
| V×N_bud_×N_tiller_ | ns | ns | ns | ns | ns | ns | ns | ** | ns | ns | ns | ns | ns | ns |
| N_main_×N_bud_×N_tiller_ | * | ns | ns | * | ns | ns | * | ns | ns | ns | ns | ns | ns | * |
| V×N_main_×N_bud_×N_tiller_ | ns | ns | ns | ns | ns | ns | ns | ns | ns | ns | ns | ns | ns | ns |

TBP, total biomass production; BP_ratoon_, biomass production during the ratoon season; CGR, crop growth rate; IR, intercepted radiation; RUE, radiation use efficiency.

ns, not significant at the 0.05 probability level; * and **, significant at the 0.05 and 0.01 probability levels, respectively.

**Table S4** Analysis of variance for biomass production, crop growth rate, leaf area index, intercepted radiation, and radiation use efficiency of ratoon crop in 2017.

| Source | TBP | Pre-BP_ratoon_ | Post-BP_ratoon_ | Total BP_ratoon_ | Pre-CGR | Post-CGR | Total CGR | LAI | Pre-IR | Post-IR | Total IR | Pre-RUE | Post-RUE | Total RUE |
| --- | --- | --- | --- | --- | --- | --- | --- | --- | --- | --- | --- | --- | --- | --- |
| Variety (V) | ** | ** | ** | ** | ** | ** | ** | ** | ** | ** | ** | ** | ns | ns |
| N_main_ | * | * | ns | * | * | ns | * | * | ns | ns | ns | * | ns | * |
| N_bud_ | ** | ** | ** | ** | ** | ** | ** | ** | * | * | * | ** | ** | ** |
| N_tiller_ | ** | ** | ** | ** | ** | ** | ** | ** | * | * | * | ** | ** | ** |
| V×N_main_ | ** | ** | ns | ** | ** | ns | ** | ns | ns | ns | ns | ** | ns | ** |
| V×N_bud_ | ** | ** | ns | * | ns | ns | ns | ** | ns | ns | ns | ns | ns | ns |
| V×N_tiller_ | ** | ** | ns | ** | ** | ns | * | ** | ns | ns | ns | ** | ns | * |
| N_main_×N_bud_ | ns | ns | ns | ns | ns | ns | ns | ns | ns | * | ns | ns | ns | ns |
| N_main_×N_tiller_ | ns | ns | ns | ns | ns | ns | ns | * | ns | * | ns | ns | ns | ns |
| N_bud_×N_tiller_ | ** | ns | ns | * | ns | ns | * | ** | ns | ** | ns | ns | ns | ns |
| V×N_main_×N_bud_ | * | ns | ns | ns | ns | ns | ns | ns | ns | ns | ns | ns | ns | ns |
| V×N_main_×N_tiller_ | ns | ns | ns | ns | ns | ns | ns | ns | ns | ns | ns | ns | ns | ns |
| V×N_bud_×N_tiller_ | ns | ns | ns | ns | ns | ns | ns | ** | ns | ns | ns | ns | ns | ns |
| N_main_×N_bud_×N_tiller_ | ns | ns | ns | ns | ns | ns | ns | ** | ns | * | ns | ns | ns | ns |
| V×N_main_×N_bud_×N_tiller_ | ns | ns | ns | ns | ns | ns | ns | * | ns | ns | ns | ns | ns | ns |

TBP, total biomass production; BP_ratoon_, biomass production during the ratoon season; CGR, crop growth rate; IR, intercepted radiation; RUE, radiation use efficiency.

ns, not significant at the 0.05 probability level; * and **, significant at the 0.05 and 0.01 probability levels, respectively.

**Table S5** Analysis of variance for N uptake and N use efficiency of ratoon crop in 2016.

| Source | TNU | NU_ratoon_ | NU_ratoon_/TNU | NUE_b_ |
| --- | --- | --- | --- | --- |
| Variety (V) | ** | ** | ** | ns |
| N_main_ | ** | ** | ns | ns |
| N_bud_ | ** | ns | * | ** |
| N_tiller_ | ** | ** | ** | ** |
| V×N_main_ | ** | ** | ** | * |
| V×N_bud_ | * | * | ns | * |
| V×N_tiller_ | ** | ** | ns | ns |
| N_main_×N_bud_ | ns | ns | ns | ns |
| N_main_×N_tiller_ | ns | ns | ns | ns |
| N_bud_×N_tiller_ | ns | ns | * | * |
| V×N_main_×N_bud_ | ns | ** | ** | * |
| V×N_main_×N_tiller_ | ns | ns | ns | ns |
| V×N_bud_×N_tiller_ | * | * | ns | ns |
| N_main_×N_bud_×N_tiller_ | ns | ns | ns | ns |
| V×N_main_×N_bud_×N_tiller_ | * | * | ns | ns |

TNU, total N uptake; NU_ratoon_, N uptake during the ratoon season; NUE_b_, N use efficiency for biomass production during the ratoon season.

ns, not significant at the 0.05 probability level; * and **, significant at the 0.05 and 0.01 probability levels, respectively.

**Table S6** Analysis of variance for N uptake and N use efficiency of ratoon crop in 2017.

| Source | TNU | NU_ratoon_ | NU_ratoon_/TNU | NUE_b_ |
| --- | --- | --- | --- | --- |
| Variety (V) | ** | ** | ** | ** |
| N_main_ | ** | ** | * | * |
| N_bud_ | ** | ** | ns | ns |
| N_tiller_ | ** | ** | ** | ** |
| V×N_main_ | * | * | ns | ns |
| V×N_bud_ | ns | ns | ** | * |
| V×N_tiller_ | * | * | ns | ns |
| N_main_×N_bud_ | ns | ns | ** | * |
| N_main_×N_tiller_ | ns | ns | ns | ns |
| N_bud_×N_tiller_ | ns | ns | ** | ns |
| V×N_main_×N_bud_ | * | ns | ns | ns |
| V×N_main_×N_tiller_ | ns | ns | ns | ns |
| V×N_bud_×N_tiller_ | ns | ns | ns | ns |
| N_main_×N_bud_×N_tiller_ | ns | ns | ns | ns |
| V×N_main_×N_bud_×N_tiller_ | ns | ns | ns | ns |

TNU, total N uptake; NU_ratoon_, N uptake during the ratoon season; NUE_b_, N use efficiency for biomass production during the ratoon season.

ns, not significant at the 0.05 probability level; * and **, significant at the 0.05 and 0.01 probability levels, respectively.
